# Stat3 is tyrosine-phosphorylated through the interleukin-6/glycoprotein 130/Janus kinase pathway in breast cancer

**DOI:** 10.1186/bcr1680

**Published:** 2007-05-25

**Authors:** Marjan Berishaj, Sizhi Paul Gao, Simi Ahmed, Kenneth Leslie, Hikmat Al-Ahmadie, William L Gerald, William Bornmann, Jacqueline F Bromberg

**Affiliations:** 1 Department of Medicine, Memorial Sloan-Kettering Cancer Center, 1275 York Avenue, New York, NY 10021 USA; 2 Laboratory of Molecular Cell Biology, 1230 York Avenue, Rockefeller University, New York, NY, 10021 USA; 3 Department of Pathology, 1275 York Avenue, Memorial Sloan-Kettering Cancer Center, New York, NY 10021 USA; 4 Division of Experimental Diagnostic Imaging, 1515 Holcombe Blvd., University of Texas M. D. Anderson Cancer Center, Houston, TX 77030 USA

## Abstract

**Introduction:**

Signal transducer and activator of transcription 3 (Stat3) is constitutively tyrosine-phosphorylated in approximately 50% of primary breast carcinomas. A number of different mechanisms responsible for Stat3 activation, including abnormal activation of receptor tyrosine kinases, Src, and Janus kinases (Jaks), have been implicated in breast cancer.

**Methods:**

We examined six breast cancer-derived cell lines expressing high or low levels of tyrosine-phosphorylated Stat3 (pStat3) as well as primary breast cancer specimens.

**Results:**

Inhibition of Src or EGFR (epidermal growth factor receptor) tyrosine kinases had no effect on pStat3 levels, whereas pan-Jak inhibitor P6 resulted in complete abrogation of Stat3 phosphorylation and inhibition of growth. Jaks are required for cytokine signaling, and the glycoprotein 130 (gp130) receptor-associated Jaks are known mediators of Stat3 phosphorylation. Blockade of the gp130 receptor or sequestration of the interleukin-6 (IL-6) ligand led to a decrease of pStat3 levels. Conditioned media from those cell lines expressing high levels of pStat3 contained IL-6 and were capable of stimulating Stat3 phosphorylation. We examined IL-6 levels in primary breast tumors and found a positive correlation between pStat3 and IL-6 expression.

**Conclusion:**

In summary, a principal mechanism of Stat3 activation in breast cancer is through the IL-6/gp130/Jak pathway.

## Introduction

The Stat (signal transducer and activator of transcription) family of proteins are transcription factors known for their role as integrators of cytokine and growth factor receptor signaling and are required for cell growth, survival, differentiation, and motility [1,2]. Stat activation is dependent upon tyrosine phosphorylation, which induces dimerization via reciprocal phosphotyrosine-SH2 (Src homology domain 2) interaction between two Stat molecules. Activated Stats translocate to the nucleus, where they bind to consensus promoter sequences of target genes and activate their transcription [3]. In normal non-transformed cells, Stat tyrosine phosphorylation is transient. However, in numerous cancer-derived cell lines and in an ever-growing number of primary tumors, Stat proteins (in particular, Stat3) are persistently tyrosine-phosphorylated [4].

Stat3 is constitutively activated in more than 50% of primary breast tumors and tumor-derived cell lines [5-17]. Clinical studies demonstrated that elevated levels of tyrosine-phosphorylated Stat3 (pStat3) in patients with stage II breast cancer correlated with an incomplete response to neo-adjuvant chemotherapy (a poor prognostic feature) [7]. The evidence that activated Stat3 participates in a causal manner in breast tumorigenesis is based on studies from cell lines expressing high levels of pStat3. Inhibiting or removing Stat3 leads to increased apoptosis, chemosensitivity, and decreased angiogenesis both in cell culture and in xenograft models [5,6,10,13,14,18-22]. Furthermore, the expression of a constitutively active form of Stat3 (Stat3C) into immortalized human breast epithelial cells mediated *de novo *tumorigenesis, demonstrating the sufficiency of this transcription factor in promoting cellular transformation [16]. Stat3 activation can occur through the actions of many autocrine and paracrine growth factors (for example, interleukin-6 [IL-6], epidermal growth factor, platelet-derived growth factor, heregulin, vascular endothelial growth factor, and hepatocyte growth factor) and the Src non-receptor tyrosine kinase, leading to the prediction that its phosphorylation in breast cancer would be through these multiple, redundantly acting growth factors [6-8,12,13,18,19,23-25]. Among these candidate cytokines, IL-6, which activates Stat3 through the glycoprotein 130/Janus kinase (gp130/Jak) pathway, is notable for its pleiotropic tumor-promoting activities, including anti-apoptotic, pro-invasive, and immune-stimulatory effects attributable to the activation of Stat3 target genes [26,27]. Furthermore, high IL-6 levels have been linked to a poor prognosis in patients with advanced breast cancer, the pathogenesis of inflammatory breast cancer, and resistance to chemotherapy [26]. Our studies have revealed a prominent and non-redundant role for IL-6 in driving Stat3 activation in breast cancer.

## Materials and methods

### Cell culture

MCF7, BT-474, MDA-MB-435, MDA-MB-468, MDA-MB-231, and MCF10A cells were purchased from the American Type Culture Collection (Manassas, VA, USA) and grown in Dulbecco's modified Eagle's medium supplemented with penicillin/streptomycin (Invitrogen Corporation, Carlsbad, CA, USA) and 10% fetal bovine serum. MCF10A cells were cultured as described previously [28]. The 4175 metastatic subline of MDA-MB-231 cells was a gift from Joan Massague (Memorial Sloan-Kettering Cancer Center [MSKCC], New York, NY, USA) and was cultured as previously described [29]. Cell proliferation was determined using an MTT (3- [4,5-dimethylthiazol-2-yl]-2,5-diphenyltetrazolium bromide) assay (as described by the vendor) with a microplate reader (Sigma-Aldrich, St. Louis, MO, USA).

### Immunohistochemical assays

Multitissue blocks of formalin-fixed, paraffin-embedded breast cancer tissue (containing three or four representative 0.6-mm cores) were prepared using a tissue arrayer, and immunohistochemistry (IHC) was performed as described [16,30]. Antigen retrieval using citric acid (pH 6.0) at 97°C for 30 minutes was followed by treatment with 3% H_2_O_2_. Phospho-Stat3 (Tyr-705) (Cell Signaling Technology, Inc., Danvers, MA, USA) was used at 1:200. Anti-human IL-6 goat polyclonal antibody (R&D Systems, Inc., Minneapolis, MN, USA) was used at a 1:100 dilution [31]. Anti-human estrogen receptor (ER) antibody (Immunotech, now part of Beckman Coulter, Fullerton, CA, USA) anti-human progesterone receptor (PR) antibody (BioGenex, San Ramon, CA, USA), and anti-Her2neu antibody (CB11; Dako North America, Inc., Carpinteria, CA, USA) were used at a 1:200 dilution. pStat3 positivity was defined as 3+, 2+, 1+, or 0 by IHC. Her2 positivity was defined as 3+ by IHC or as 2+ by IHC with a gene amplification of 2.1 or greater [30]. For ER and PR, samples were considered positive if more than 10% of cell nuclei were immunoreactive. Scoring of the tissue microarray was performed by two independent observers (JFB and MB) with a high correlation between scorers (*p *< 0.001) for both pStat3 and IL-6. For a tumor to be considered positive for either pStat3 or IL-6, all four replicates in the tissue array had to have a similar staining intensity; otherwise, the tumor was excluded. Statistical analyses were performed using STATVIEW (SAS Institute Inc., Cary, NC, USA). The correlation between the scores of both scorers and the relationship between that of pStat3 and IL-6, ER, PR, and Her2neu were measured using the χ^2 ^test.

### Enzyme-linked immunosorbent assay

Cells were grown to approximately 50% to 85% confluence, and conditioned media (CM) was analyzed for IL-6 by means of an IL-6 enzyme-linked immunosorbent assay (ELISA) kit (Cell Sciences, Inc., Canton, MA, USA) according to the manufacturer's instructions.

### Western blot and antibodies

Whole-cell, nuclear, and cytoplasmic extracts were prepared as previously described [5]. Protein concentration was determined using the Bradford assay (Bio-Rad Laboratories, Inc., Hercules, CA, USA), and Western blot analysis was carried out by standard methods. Antibodies for Western blotting included Stat3, pStat3, Src, and pSrc (Cell Signaling Technology, Inc.). Phosphorylated Jak2 and Jak2 antibodies were obtained from Calbiochem (San Diego, CA, USA) and Upstate (now part of Millipore Corporation, Billerica, MA, USA), respectively. Gp130-blocking antibody, BR-3, and IL-6-blocking antibody, 522, were obtained from Cell Sciences, Inc. Leukemia inhibitory factor (LIF)-blocking and oncostatin M (OSM)-blocking antibodies were purchased from R&D Systems, Inc.

### Reagents

Tetracyclic pyridone 2-*tert*-butyl-9-fluoro-3,6-dihydro-7*H*-benz [h]-imidaz [4,5-*f*]isoquinoline-7-one, P6, was synthesized by author WB [37]. Gefitinib (ZD) was a gift from AstraZeneca (London, UK) provided by Jackie She from the Anti-Tumor Assessment Core Facility, MSKCC. Dasatinib (BMS-354825) was synthesized by Darren Veach at MSKCC.

## Results

### Stat3 is tyrosine-phosphorylated in approximately 50% of primary breast tumors

**Figure 1:**
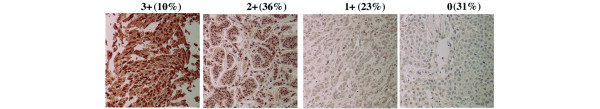
Signal transducer and activator of transcription 3 (Stat3) phosphorylation in primary breast cancer. Tissue microarrays of primary breast tumors (85) were analyzed for nuclear tyrosine-phosphorylated Stat3 (pStat3) by immunohistochemical analysis. Ten percent expressed high levels (3+), 36% moderate levels (+2), 23% low levels (+1), and 31% no detectable pStat3 (0).

Persistently activated Stat3 has been shown to play an important role in the pathogenesis of breast tumorigenesis based primarily on studies in breast cancer-derived cell lines using anti-sense, short interfering RNA and dominant negative Stat3 constructs and a variety of different drugs leading to Stat3 inhibition. The mechanisms of Stat3 activation have been presumed to involve multiple, redundantly acting growth factors and activated tyrosine kinases, making inhibition of one of these unlikely to lead to effective inhibition of Stat3 in breast cancer. To explore these potential mechanisms, we evaluated 85 primary breast cancer specimens and examined nuclear pStat3 levels by IHC. We determined that 46% expressed high to moderate levels (3, 2+) of nuclear pStat3, 23% low levels (+1), and 31% no detectable pStat3 (Figure 1). Breast tumors are classified by expression of the nuclear hormone receptors estrogen and progesterone as well as the receptor tyrosine kinase Her2neu principally as a consequence of the available therapeutics targeting these pathways (selective ER modulators, aromatase inhibitors, trastuzumab, and laptinib). We also examined ER, PR, and Her2neu levels by IHC, and no correlation consistent with prior reports was observed between high to moderate nuclear pStat3 and high ER, PR, or Her2neu expression (Table 1) [7,11,17].

**Table 1:** Immunohistochemical analysis of 85 primary breast tumors for pStat3, interleukin-6, estrogen receptor, progesterone receptor, and Her2neu

	pStat3 0, 1+ (54%)	pStat3 2, 3+ (46%)	*P *value (Fisher)
Interleukin-6 (2, 3+)	11	32	0.001
Interleukin-6 (0, 1+)	34	5	
Estrogen receptor-positive	21	25	0.20
Estrogen receptor-negative	24	14	
Progesterone receptor-positive	16	18	1.0
Progesterone receptor-negative	29	19	
Her2neu (2, 3+)	5	7	1.0
Her2neu (0, 1+)	30	25	

The correlations between the relationship of pStat3 and interleukin-6, estrogen receptor, progesterone receptor, and Her2neu were measured using the χ^2 ^test. 0 is no staining, 1+ is weak staining, 2+ is moderate staining and 3+ is strong staining for pStat3, tyrosine-phosphorylated signal transducer and activator of transcription 3 and IL-6.

### Stat3 is constitutively tyrosine-phosphorylated in breast cancer-derived cell lines

**Figure 2:**
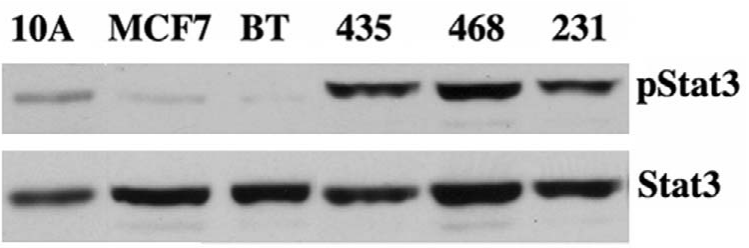
Differential signal transducer and activator of transcription 3 (Stat3) phosphorylation in breast cancer-derived cell lines. Nuclear extracts (20 μg) isolated from MCF10A, MCF7, BT474, MDA-MB-435, MDA-MB-MD-468, and MDA-MB-MD-231 cell lines were analyzed by Western blotting with tyrosine-phosphorylated Stat3 (pStat3) and Stat3 antibodies.

We examined the relative levels of pStat3 in an immortalized breast epithelial cell line as well as five different breast cancer-derived cell lines. MCF10A, MCF7, and BT474 cell lines had low levels of pStat3, whereas MDA-MB-435, MDA-MB-468, and MDA-MB-231 cell lines expressed higher levels of pStat3 (Figure 2) [10,12,13]. We also examined tyrosine-phosphorylated Stat1 and found that only the MDA-MB-468 cells contained low levels of pStat1 (data not shown).

### Stat3 phosphorylation is blocked by Jak kinase inhibition but not Src or epidermal growth factor receptor blockade

**Figure 3:**
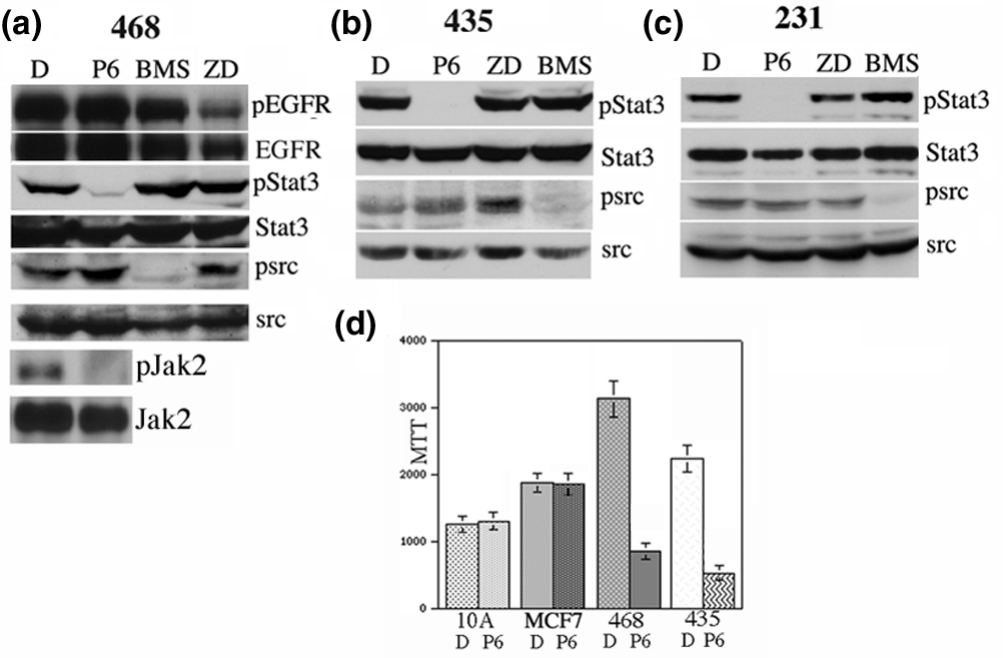
Signal transducer and activator of transcription 3 (Stat3) phosphorylation is mediated through Janus kinases (Jaks) but not epidermal growth factor receptor (EGFR) or Src kinases. **(a) **Radioimmunoprecipitation assay (RIPA) extracts (50 μg) isolated from MDA-MB-468 cells treated for 4 hours with dimethyl sulfoxide (DMSO), P6 1 μM, BMS 50 nM, and ZD 2 μM were analyzed for pEGFR, total EGFR, tyrosine-phosphorylated Stat3 (pStat3), Stat3, pSrc, Src, pJak2, and Jak2. **(b) **Nuclear extracts (20 μg) isolated from MDA-MB-435 cells (upper panels) and RIPA extracts (bottom panels) treated as in **(a) **and probed with the indicated antibodies. **(c) **Nuclear extracts (20 μg) isolated from MDA-MB-231 cells (upper panels) and RIPA extracts (bottom panels) treated as in **(a) **and probed with the indicated antibodies. **(d) **Ten thousand cells (MCF10A, MCF7, MDA-MB-468, and MDA-MB-435) were plated into a 96-well culture dish and treated with DMSO control or P6 (1 μM) for 48 hours, and proliferation was measured using an MTT (3- [4,5-dimethylthiazol-2-yl]-2,5-diphenyltetrazolium bromide) assay.

A number of different tyrosine kinases, including the epidermal growth factor receptor (EGFR) family of tyrosine kinases, Src, and Jaks, have been described as mediating Stat3 phosphorylation in both primary tumors and breast cancer-derived cell lines [6,13,23,25,32]. Specifically, it has been demonstrated that an inhibitor of Src kinase resulted in abrogation of pStat3 in the above-described cell lines and that a positive correlation between pStat3 and pSrc existed in primary breast cancer samples [6,13]. Similarly, an inhibitor of EGFR was shown to inhibit Stat3 activation and it was shown that high EGFR was positively correlated with Stat3 in breast cancer [8,19,23,24,32]. The gold standard for Jak2 inhibition has been the tyrphostin AG490 [33-35]. We recently compared the sensitivity and specificity of AG490 to those of a novel pan-Jak inhibitor (P6) on IL-6-dependent myeloma cells and determined that P6 inhibited Jak/Stat3 signaling with higher sensitivity and specificity than those of AG490, which indiscriminately inhibited growth of cell lines, even those lacking activated Jaks and Stats [35-37]. Here, we examined the effects of a pan-Jak inhibitor (P6), a potent inhibitor of Src (dasatinib, BMS), and an EGFR/Her2neu inhibitor (Gefitinib, ZD) on pStat3 in these breast cancer-derived cell lines [13,36,38-40]. We observed no effect of either the Src or EGFR inhibitors on pStat3 in MDA-MB-435, MDA-MB-468, and MDA-MB-231 cells, whereas the pan-Jak inhibitor led to a profound decrease in pStat3 levels in all cell lines examined (Figure 3). Low levels of pJak2 were observed in the MDA-MB-468 cells, which were inhibited by P6 (Figure 3a). Decreased pSrc and pEGFR was noted in the BMS- and ZD-treated cells, respectively, demonstrating the efficacy of these drugs. Thus, in contrast to other reports, we did not observe any effect of either EGFR/Her2 or Src inhibition on Stat3 activation in breast cancer-derived cell lines [13,19,32]. However, in all cases, Jak inhibition resulted in complete abrogation of pStat3 levels. We examined the growth of MDA-MB-435, MDA-MB-468, MCF-7, and MCF10A cells in the presence and absence of P6 and observed growth inhibition by P6 of the cancer-derived cell lines expressing high levels of pStat3 (Figure 3d).

### Gp130 and IL-6 blockade inhibits Stat3 activation in breast cancer-derived cell lines

**Figure 4:**
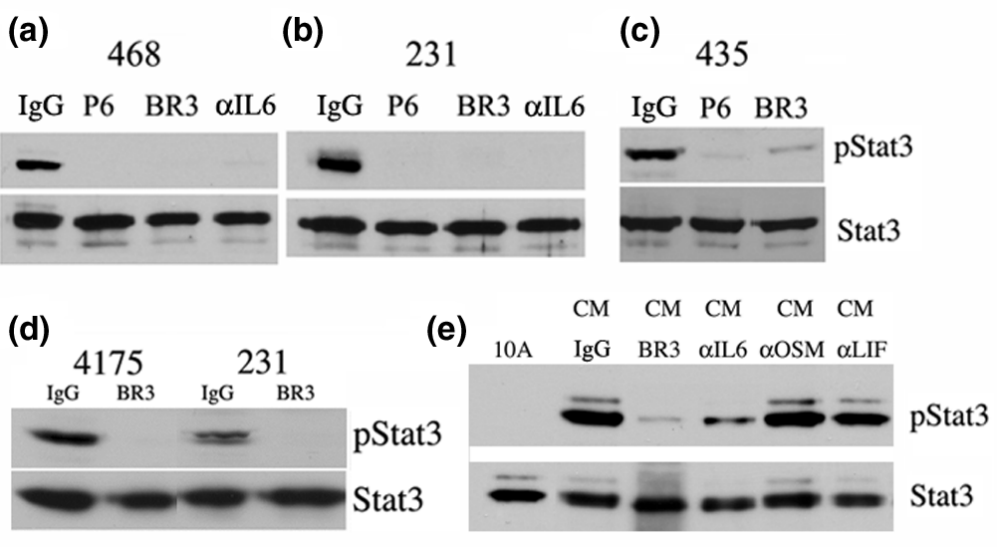
Signal transducer and activator of transcription 3 (Stat3) activation is through the interleukin-6/glycoprotein 130/Janus kinase (IL-6/gp130/Jak) pathway. **(a) **Nuclear extracts isolated from MDA-MB-468 (468) cells treated with immunoglobulin G (IgG) (10 μg/ml), P6 (1 μM), BR3 (10 μg/ml), and α-IL-6 (1 μg/ml) for 16 hours were analyzed for tyrosine-phosphorylated Stat3 (pStat3) and total Stat3 protein. **(b) **Nuclear extracts isolated from MDA-MB-231 (231) cells treated with IgG (10 μg/ml), P6 (1 μM), BR3 (10 μg/ml), and α-IL-6 (1 μg/ml) for 16 hours were analyzed for pStat3 and total Stat3. **(c) **Nuclear extracts isolated from MDA-MB-435 (435) cells treated with IgG (10 μg/ml), P6 (1 μM), and BR3 (10 μg/ml) for 16 hours were analyzed for pStat3 and total Stat3. **(d) **Nuclear extracts isolated from 4175 and 231 cells treated with IgG (10 μg/ml) and BR3 (10 μg/ml) for 16 hours were analyzed for pStat3 and total Stat3. **(e) **Nuclear extracts isolated from MCF10A cells treated for 30 minutes with conditioned media (CM) from MDA-MB-468 cells after pretreatment for 4 hours with IgG control antibody, BR3 (5 μg/ml), α-IL-6 antibody (5 μg/ml), α-oncostatin M (OSM) antibody (10 μg/ml), and α-leukemia inhibitory factor (LIF) antibody (10 μg/ml) were analyzed for pStat3 and total Stat3.

Jaks are obligate mediators of cytokine receptor signaling. Among these candidate cytokines, IL-6, which activates Stat3 through the gp130/Jak pathway, is notable for its pleitrophic tumor-promoting activities, including anti-apoptotic, pro-invasive, and immune-stimulatory effects attributable to the activation of Stat3 target genes [26,27]. We hypothesized that Jaks (Jak1, Jak2, and Tyk2) in association with the gp130 receptor were the principal mediators of Stat3 activation in these cell lines. Blockade of the gp130 receptor led to a decrease in pStat3 levels in MDA-MB-435, MDA-MB-468, and MDA-MB-231 cells (Figure 4). Sequestration of the IL-6 ligand using an anti-human IL-6 antibody also led to a significant reduction in pStat3 levels in MDA-MB-231, 4175, and MDA-MB-468 cell lines (Figure 4 and data not shown). Recently, individual single-cell-derived isolates subcloned from the breast cancer cell line MDA-MB-231 were characterized as having distinct metastatic abilities and tissue tropisms [41]. Specifically, the 4175 subline metastasizes to the lung when injected into the tail vein whereas the parental 231 line does not [41]. We examined the relative levels of pStat3 in the parental 231 cells and 4175 subline and determined that pStat3 levels were higher in the highly metastatic 4175 line (Figure 4d). Blockade of the gp130 receptor inhibited Stat3 phosphorylation in the 4175 subline as well as the parental 231 cell line (Figure 4d). We confirmed in these cultured cells that autocrine production of IL-6 is directly responsible for activating Stat3. CM from MDA-MB-468, MDA-MB-231, and 4175 cell lines contained high levels of IL-6 as measured by ELISA (942, 2,456, and 6,216 pg/ml, respectively). Furthermore, activation of Stat3 in MCF10A cells elicited by CM from MDA-MB-468 cells could be blocked by both the gp130-blocking antibody and the IL-6 sequestering antibody, but not by anti-OSM or anti-LIF antibodies (Figure 4e). Similar results were obtained using CM from MDA-MB-231 and 4175 cells (data not shown). These data suggest that the mechanism of Stat3 activation in these breast cancer-derived cell lines is through the IL-6 family of cytokines, which are acting in an autocrine/paracrine manner.

### High IL-6 and pStat3 levels positively correlate in primary breast cancer

**Figure 5:**
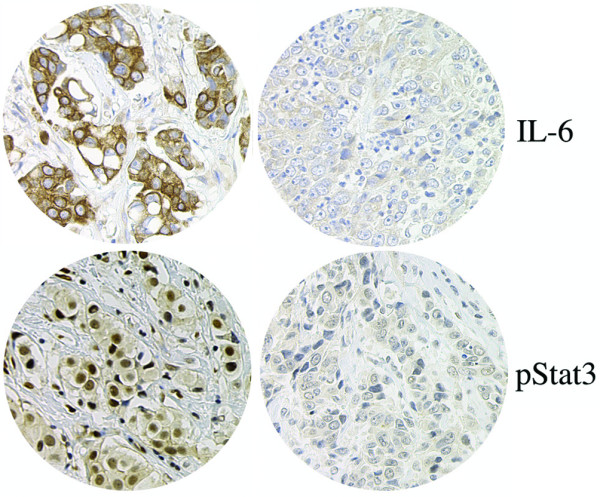
Immunohisotchemical analaysis of primary breast cancer specimens for interleukin-6 (IL-6) (upper panels) and tyrosine-phosphorylated signal transducer and activator of transcription 3 (pStat3) (bottom panels). Examples of high pStat3 and IL-6 (left panels) as well as low pStat3 and IL-6 (right panels) are indicated.

To directly examine the singular importance of IL-6 as the activator of Stat3 in breast cancer, pStat3 levels and IL-6 levels were compared by IHC analysis of our primary breast cancer specimens (Figure 1). Eighty-two were evaluable for IL-6, and 52% of these expressed moderate to high levels of IL-6 (2, 3+) whereas 48% expressed low levels of IL-6. Examples of both high and low IL-6 by IHC are shown in Figure 5. A positive correlation between (2, 3+) pStat3 and (2, 3+) IL-6 levels was observed (*p *< 0.001) (Table 1).

## Discussion

By using animals deficient for Stat3 in certain tissues or by introducing dominant negative Stat3 constructs and anti-sense and short hairpin RNA Stat3 molecules into cell lines, many investigators have demonstrated a requirement for Stat3 in tumorigenesis [42]. Thus, inhibiting Stat3 activity or expression is likely to be an important therapeutic modality for a number of malignancies, including breast cancer. Targeting the Stat3 molecule directly is of significant interest, and indeed several small molecules that appear to target the Stat3 SH2 domain or the DNA-binding capacity of Stat3 have been developed [43-45]. It has been assumed that the kinases responsible for Stat3 activation are many, and therefore targeting one kinase or receptor would not be of benefit for the treatment of a variety of tumors.

Here, we examined the mechanisms of Stat3 phosphorylation in breast cancer. We observed that high pStat3 levels in primary tumor specimens did not correlate with nuclear hormone receptor status (ER or PR) nor overexpression of the Her2neu receptor (Table 1). It has been suggested in murine models of breast cancer that aberrant Her2neu signaling may mediate Stat3 activation [22]. However, in primary human breast tumors, no such relationship was observed [7,11,17]. We examined pSrc and pEGFR levels by IHC in these tumor specimens, but less than 3% of the samples had elevated expression of these proteins, possibly for technical reasons as these values appear to be too low compared to other reports (data not shown) [7,8]. To examine the potential mechanisms involved in mediating Stat3 phosphorylation, we examined breast cancer-derived cell lines expressing high levels of pStat3. Inhibition of EGFR and Src kinases did not have an effect on pStat3, suggesting that these kinases are not direct mediators of Stat3 phosphorylation in these breast cancer-derived cell lines. Our data regarding Src inhibition differ from those described by Garcia and colleagues [13] whereby treatment of MDA-MB-468 cells with an Src inhibitor (PD180970) resulted in inhibition of pStat3. Similarly, it was reported that treatment of MDA-MB-231 cells with an EGFR inhibitor (AG1478) inhibited Stat3 activation [19]. In our study, we used different inhibitors of both Src and EGFR at concentrations previously described to potently inhibit both of these kinases. The explanations for these discrepancies are not clear, but differences in inhibitor selectivity and the length of time for which cells are treated could explain our differing observations. Interestingly, dasatinib treatment of both lung and prostate cancer-derived cell lines did not affect pStat3 levels, suggesting that Src inhibition does not play a role in mediating Stat3 activation in these cancer-derived cell lines [38,39]. In contrast to EGFR and Src inhibition, a pan-Jak inhibitor inhibited pJak2 and Stat3 phosphorylation (Figure 3). Furthermore, treatment of the cell lines with an anti-gp130 antibody significantly inhibited Stat3 phosphorylation (Figure 4). Given the previously described association between the EGFR family members and gp130 receptor, we attempted but were unable to show any physical association between EGFR and gp130 by immunoprecipitation experiments in MDA-MB-468 cells (which express the highest levels of EGFR) (data not shown) [19].

The cytokine receptor gp130 is the common signaling subunit for the IL-6 family of cytokines. The gp130 receptor is ubiquitously expressed in tissues and mediates numerous homeostatic functions, including immune response, inflammation, bone metabolism, and hematopoiesis [27]. IL-6, IL-11, OSM, LIF, and ciliary neurotropic factor are among the IL-6 family cytokines that use and require gp130 as a signaling subunit [27]. We determined that IL-6 was the essential cytokine mediating gp130 activation in MDA-MB-468 and MDA-MB-231 cells and the 4175 subline (Figure 4). We did not observe inhibition of pStat3 by the IL-6-blocking antibody in the MDA-MB-435 cell line nor was IL-6, LIF, or OSM detected in the CM from this cell line. We believe that IL-11 is responsible for mediating Stat3 activation because IL-11 mRNA levels were increased in this cell line compared to MDA-MB-468 cells (data not shown). However, we have yet to confirm by immunological means that IL-11 is mediating gp130 activation.

The data presented here suggest that the principal mediator of Stat3 activation in breast cancer is through the IL-6 family of cytokines activating the gp130/Jak signaling pathway. Stat3 activation through the gp130 receptor is regulated by both positive effectors such as IL-6 and negative regulators, including suppressor of cytokine signaling (SOCS) 1 and SOCS3, which inhibit Jak activity [46]. SOCS1 and SOCS3 promoters are frequently methylated in primary tumors and cancer-derived cell lines, resulting in decreased expression of these negative regulators of Jaks and leading to sustained Jak activation [47]. We hypothesize that even in the absence of IL-6 or IL-6 engagement with the gp130 receptor, Jaks may still be activated due to the lack of SOCS gene expression and can only be fully inhibited with a Jak inhibitor.

To directly examine the singular importance of IL-6 as the activator of Stat3 in breast cancer, pStat3 levels and IL-6 levels were compared by IHC analysis of 82 primary breast cancer specimens and a significant positive correlation was observed between high pStat3 and high IL-6 levels as determined by IHC (*p *< 0.001). We also examined levels of gp130 and were unable to obtain consistent immunohistochemical data (data not shown).

## Conclusion

We propose that the IL-6/gp130/Jak signaling pathway plays a critical role in Stat3 activation in human breast cancer and that blockade of this pathway may be an important therapeutic modality in breast cancer.

## Abbreviations

CM = conditioned media; EGFR = epidermal growth factor receptor; ELISA = enzyme-linked immunosorbent assay; ER = estrogen receptor; gp130 = glycoprotein 130; IHC = immunohistochemistry; IL = interleukin; Jak = Janus kinase; LIF = leukemia inhibitory factor; MSKCC = Memorial Sloan-Kettering Cancer Center; OSM = oncostatin M; PR = progesterone receptor; pStat3 = tyrosine-phosphorylated signal transducer and activator of transcription 3; SH2 = Src homology domain 2; SOCS = suppressor of cytokine signaling; Stat3 = signal transducer and activator of transcription 3.

## Competing interests

The authors declare that they have no competing interests.

## Authors' contributions

MB carried out the majority of the experiments and drafted the manuscript. SA conceived a critical experiment. SPG and KL each performed experiments and contributed to several figures. HA and WLG generated the necessary tissue microarrays. WB synthesized the Jak inhibitor. JFB conceived of the study, participated in its design and coordination, and helped draft the manuscript. All authors read and approved the final manuscript.
